# 

**Published:** 1929

**Authors:** 


### The Medical Library of the University of Bristol,

WITH WHICH IS INCORPORATED

### The Library of the Bristol Medico-Chirurgical Society.

---

*The following donations have been received since the publication of the list in July,* 1929.

October, 1929.

| | | |
|---|---|---|
| American Laryngological Association (1) | .. | 1 volume. |
| College of Physicians of Philadelphia (2) | .. | 1 ,, |
| Professor E. Fawcett (3) | .. .. .. | 1 pamphlet. |
| Professor E. W. Hey Groves (4) | .. .. | 11 volumes. |
| Librarian, Armstrong College, Newcastle-on-Tyne (5) | .. .. .. .. .. | 1 pamphlet. |
| Rockefeller Foundation (6) | .. .. .. | 1 volume. |
| Surgeon-General of U.S. Army (7) | .. .. | 3 volumes. |
| United Fruit Company (8) | .. .. .. | 1 volume. |
| University of Otago Medical School (9) | .. | 1 ,, |
| University of Toronto (10) | .. .. .. | 1 ,, |

Unbound periodicals have also been received from Professor E. W. Hey Groves.

### THE ONE HUNDRED AND TWENTY-NINTH LIST OF BOOKS.

The figures in round brackets refer to the figures after the names of the donors. The books to which no such figures are attached have either been bought from the Library Fund or received through the *Journal.*

| | | | |
|---|---|---|---|
| **Behrend, M.** | *Die Vor- und Nachbehandlung bei chirurgischen Eingriffen* .. .. .. .. .. .. .. .. | (4) | 1929 |
| **Burnet, J.** | *Diseases of the newborn* .. .. .. .. .. .. .. .. .. .. | | 1927 |
| **Butler, T. H.** | *Illustrated guide to the slit-lamp* .. .. .. .. .. | | 1927 |
| **Buzzard, Sir E. F.** | *Outline of Neurology and its Outlook* .. .. | (5) | 1929 |
| *Cancer and Cancer Research* .. .. .. .. .. .. .. .. .. .. .. .. .. .. | | | 1928 |
| *Catalogue of Lewis's Library to the end of* 1927. Part I. .. .. .. .. .. | | | 1929 |
| **Charleton, R., and others.** | *Four Treatises on the Bath Waters* (bound in 1 vol.) .. .. .. .. .. .. .. .. .. .. | | 1751–54 |
| **Clarke, E.** | *Refraction of the Eye* .. .. .. .. .. | 5th Ed. | 1924 |
| **Cope, V. Z.** | *Treatment of the Acute Abdomen* .. .. .. .. .. | | 1926 |
| **Cope, V. Z.** | *Some Principles of Minor Surgery* .. .. .. | | 1929 |
| **Culpin, M.** | *Medicine ; and the Man* .. .. .. .. .. .. .. .. .. | | 1927 |
| **Daukes, S. H.** | *The Medical Museum* .. .. .. .. .. .. .. .. .. | | 1929 |

| | | | |
|---|---|---|---|
| **Douthwaite, A. H.** | *Injection Treatment of Varicose Veins* 4th Ed. | (4) | 1929 |
| **Dufourmental, L.** | *Chirurgie de l'articulation temporo-maxillaire* | (4) | 1929 |
| **Freund, E.** | *Gelenkerkrankungen* | (4) | 1929 |
| **Friel, A. R.** | *Notes on Chronic Otorrhœa* | | 1929 |
| **Ker, C. B.** | *Infectious Diseases* | 3rd Ed. | 1929 |
| **Kurtzahn, H.** | *Kleine Chirurgie* | (4) | 1929 |
| **Lord, J. R., Ed.** | *The Mott Memorial* | | 1929 |
| **McLatchie, J. D. P.** | *Varicose Veins* | | 1928 |
| **Meagher, E. T.** | *General Paralysis and its Treatment by Induced Malaria* | (3) | 1929 |
| *Medical Department of United States Army in the World War.* Vols. III., X., XII. | | (7) | 1929 |
| **Naegeli, T.** | *Graphic Guide to Elementary Surgery* | | 1929 |
| **Néeris, G. R.** | *Le tractus thyréoglosse* | (4) | 1929 |
| **Ogilvie, W. H.** | *Recent Advances in Surgery* 2nd Ed. | (4) | 1929 |
| **Oudard, P., and others.** | *Le diagnostic dans les affections de la colonne vertébrale* | (4) | 1928 |
| **Overend, W.** | *Radiography of the Chest.* II. | | 1928 |
| **Percival, A. S.** | *The Prescribing of Spectacles* | 3rd Ed. | 1928 |
| *Praktische Differentialdiagnose.* Hrsg. von G. Honigmann. Bd. IV., Chirurgie | | (4) | 1928 |
| **Ray, M. B.** | *On Prescribing Physical Treatment* | | 1929 |
| **Stephenson, T.** | *Incompatibility in Prescriptions* | New Ed. | 1929 |
| **Troup, W. A.** | *Ultra-Violet Rays in General Practice* | | 1926 |
| **Weeks, C. C.** | *Alcohol and Human Life* | | 1929 |

TRANSACTIONS, REPORTS, JOURNALS, ETC.

| | | |
|---|---|---|
| Collected Papers of the Mayo Clinic. Vol. XX., 1928 | | 1928 |
| Methods and Problems of Medical Education. 13th Series | (6) | 1929 |
| Modern Technique in Treatment. Vol. IV. | (4) | 1928 |
| Proceedings of the University of Otago Medical School. No. 6 | (9) | 1929 |
| Selected articles, Department of Pediatrics, University of Toronto | (10) | 1929 |
| Surgical Clinics of North America. June, 1929 | | 1929 |
| Transactions of the American Laryngological Association. Vol. 50 | (1) | 1928 |
| Transactions of the College of Physicians, Philadelphia | (2) | 1929 |
| United Fruit Company, 17th Annual Report | (8) | 1928 |

### Local Medical Notes

APPOINTMENTS.

UNIVERSITY OF LONDON.—Mr. L. E. Claremont, M.R.C.S., L.R.C.P., L.D.S., has been appointed Director of the Eastman Clinic, Royal Free Hospital, London.

UNIVERSITY OF SINGAPORE.—Mr. E. K. Tratman, B.D.S., L.D.S., has been appointed Professor of Dental Surgery and Director of Dental Studies.

T

ROYAL COLLEGE OF PHYSICIANS, LONDON.—Dr. Carey F. Coombs has been appointed Lumleian Lecturer for 1930.

UNIVERSITY OF WALES.—Robert Victor Cooke, M.B., Ch.B. Bristol, F.R.C.S. Eng., has been appointed Junior Assistant to the Surgical Unit of the National School of Medicine, Cardiff.

### EXAMINATION RESULTS.

Students of the University of Bristol have recently passed the following examinations :—

UNIVERSITY OF LONDON. — M.B., B.S.—K. H. Pridie. *Group I. only,* L. E. Vine. *Second Examination* (*Part I.*), H. F. M. Finzel. (*Part II.*), A. L. Eyre-Brook.

R.C.S. ENG., *Primary* F.R.C.S.—D. L. Eyre-Brook. L.D.S., R.C.S.—*Pass :* R. G. Boodle. *Final Examination* (*Part I.*). *Pass :* Q. A. Davies, M. E. Minton, A. H. V. Williams.

R.C.P. LONDON and R.C.S. ENGLAND.—*Diploma in Tropical Medicine and Hygiene,* A. H. Lowther, M.B., Ch.B. (Bristol).

CONJOINT BOARD.—M.R.C.S., L.R.C.P.—*Pass : In Anatomy,* W. R. Cambridge. *In Physiology,* J. F. H. Eagles. *In Pharmacology and Materia Medica,* J. L. S. James, W. E. Thomas. *In Medicine,* *C. E. Brierley, *N. D. Gerrish, *J. J. J. Giraldi, N. L. Price. *In Surgery,* *J. J. J. Giraldi, N. L. Price, L. H. Wharton, J. E. Newton, *R. G. Mayer, *W. H. B. Spoor, R. M. Ealand. *In Midwifery,* *J. J. J. Giraldi, C. H. Pauli, R. Margaret Hickman, J. E. Newton, W. E. Thomas.

L.S.A.—*In Surgery,* T. M. White. *In Surgery and Medicine,* N. H. Kettlewell (granted diploma).

* Qualified.

### LONG FOX MEMORIAL LECTURE.

The Long Fox Memorial Lecture was delivered by Mr. A. Rendle Short, B.Sc., M.D., F.R.C.S., in the Physiological Lecture Theatre of the University of Bristol on Tuesday, October 22nd, 1929. Subject: "Ten Years' Progress in Surgical Treatment."

